# Transcriptomic Profiling Identifies Disease-Specific miRNA–mRNA Regulatory Networks in Systemic Sclerosis

**DOI:** 10.3390/biom16070994

**Published:** 2026-07-07

**Authors:** Dóra Csige, János Rózsa, Monika Bodoki, Dóra Tari, Zsuzsanna Gyetkó, Zsófia Hagymási-Szabó, Ferenc Tóth, János Kádas, Zoltán Szekanecz, Gabriella Szűcs, Szilvia Szamosi, Szilárd Póliska, Levente Bodoki

**Affiliations:** 1 Division of Rheumatology, Department of Internal Medicine, Faculty of Medicine, University of Debrecen, 4032 Debrecen, Hungary; bodoki.monika@med.unideb.hu (M.B.); tari.dora@med.unideb.hu (D.T.); gyetko.zsuzsanna@med.unideb.hu (Z.G.); szekanecz.zoltan@med.unideb.hu (Z.S.); szucs.gabriella@med.unideb.hu (G.S.); szamosi.szilvia@med.unideb.hu (S.S.); 2 Doctoral School of Clinical Medicine, University of Debrecen, 4032 Debrecen, Hungary; 3 Genomic Medicine and Bioinformatics Core Facility, Department of Biochemistry and Molecular Biology, Faculty of Medicine, University of Debrecen, 4032 Debrecen, Hungary; rozsa.janos@ud-genomed.hu (J.R.); poliska@med.unideb.hu (S.P.); 4 UD-GenoMed Medical Genomic Technologies Ltd., 4032 Debrecen, Hungary; szabo.zsofia@med.unideb.hu (Z.H.-S.); toth.ferenc@ud-genomed.hu (F.T.); kadasj@med.unideb.hu (J.K.); 5 Doctoral School of Molecular Cell and Immune Biology, University of Debrecen, 4032 Debrecen, Hungary

**Keywords:** systemic sclerosis, biomarkers, gene expression, miRNAs

## Abstract

Systemic sclerosis (SSc) is a severe autoimmune rheumatic disease with high mortality. Epigenetic factors, particularly micro-RNAs (miRNAs), may contribute to its pathogenesis by regulating gene expression. In this cross-sectional study, we assessed altered miRNA–mRNA regulatory networks in SSc and associated them with disease-related biological processes. We analyzed the miRNA profiles and differentially expressed genes (DEGs) of peripheral blood mononuclear cells (PBMCs) from 52 SSc patients (42 women and 10 men; mean age: 59.1 years) and 24 age- and gender-matched healthy controls. Total RNA was isolated and subjected to high-throughput next-generation sequencing for both miRNA and mRNA profiling. We identified 58 differentially expressed miRNAs (DEMs), 33 upregulated and 25 downregulated in SSc. In parallel, 6610 DEGs were detected (Mann–Whitney U-test, *p* < 0.05); 31 remained upregulated and nine downregulated after false discovery rate (FDR) correction. Integration of miRNA and mRNA data revealed 180 validated inverse miRNA–mRNA interactions. Notably, 22 of 31 upregulated DEGs corresponded to targets of downregulated miRNAs, indicating coordinated derepression. Functional enrichment analyses highlighted pathways related to extracellular matrix (ECM) remodeling, immune responses, fibrosis, and transcriptional regulation. Our findings suggest that altered miRNA expression contributes to widespread transcriptional dysregulation in SSc, promoting pro-fibrotic and immune-activated molecular pathways through coordinated miRNA–mRNA interactions.

## 1. Introduction

Systemic sclerosis (SSc) is a clinically heterogeneous multisystem autoimmune disorder, defined by a distinct triad of immune dysregulation, extensive microvascular damage, and progressive visceral and cutaneous fibrosis. Based on the extent of skin involvement and severity of manifestations, SSc is typically classified into limited cutaneous (lcSSc) and diffuse cutaneous (dcSSc) subtypes, according to the established classification criteria [1]. dcSSc involves extensive skin fibrosis and is commonly linked to early and severe internal organ involvement, whereas lcSSc is characterized by distal skin sclerosis restricted to areas below the elbows and knees and the clavicular region, typically exhibiting a milder course and less pronounced visceral manifestations [1]. Prior work has shown that circulating miRNA patterns can discriminate between dcSSc and lcSSc, supporting the idea that different SSc subsets have distinct post-transcriptional regulatory programs [2].

SSc has one of the highest disease-specific mortality rates among systemic autoimmune diseases, mainly due to irreversible organ damage such as interstitial lung disease (ILD) and pulmonary arterial hypertension (PAH) [3,4,5,6,7]. Despite extensive research, the molecular and cellular mechanisms driving SSc pathogenesis remain incompletely understood, creating diagnostic and therapeutic challenges [8,9]. ILD and PAH are among the most severe internal organ complications of SSc and major contributors to mortality. SSc-ILD arises from the interplay of immune activation, vascular dysfunction, and progressive fibrosis, leading to impaired gas exchange and functional decline, whereas SSc-PAH has been suggested to represent a distinct molecular entity with specific transcriptional signatures [10,11,12,13,14,15,16]. In particular, patients with dcSSc are at increased risk of rapid pulmonary function decline, highlighting the need for molecular markers that can support early identification and monitoring of progressive disease [11,17]. In this context, miRNAs have emerged as important post-transcriptional regulators of gene expression and may provide insight into unresolved molecular pathways underlying SSc pathogenesis [18,19]. Given the availability of individual miRNAs to regulate multiple target genes, integrated miRNA–mRNA profiling may help identify regulatory networks involved in inflammation and fibrosis. However, such integrated analyses in PBMCs from SSc patients remain limited, highlighting the need for further transcriptomic studies [19,20,21,22]. PBMCs, including lymphocytes and monocytes, play central roles in immune responses and are readily accessible through minimally invasive blood sampling [23,24]. This makes PBMCs suitable for transcriptomic profiling and longitudinal monitoring, particularly when tissue biopsies are not available. As these immune cells continuously circulate through all affected organs, they potentially harbor the molecular imprints of the ongoing systemic inflammation and vascular injury, potentially preceding the clinical manifestation of irreversible fibrosis. PBMC-associated miRNAs are detectable in blood samples and may provide insight into circulating immune-cell regulatory mechanisms, making them promising candidates for clinical application [25,26]. Despite their biological relevance, miRNA regulatory landscapes in PBMCs have not yet been comprehensively characterized in SSc, leaving a significant gap in our understanding of the epigenetic drivers of the disease.

### Objectives

To address this knowledge gap, the present study employed high-throughput miRNA and mRNA sequencing in PBMCs obtained from SSc patients and matched healthy controls, as well as an integrated bioinformatics approach, to identify altered miRNA–target networks in SSc-derived PBMCs, aiming to uncover novel molecular targets that contribute to the complex pathogenesis of the disease.

ILD and PAH are major determinants of the prognosis in SSc and remain the leading causes of SSc-related mortality, underscoring the need for improved early diagnostic and monitoring strategies.

## 2. Materials and Methods

### 2.1. Study Population

Fifty-two patients were included in this study consecutively, while attending their 3–6 months follow-up in the Department of Rheumatology and Immunology, Clinical Centre, University of Debrecen, Hungary. Additionally, 24 age- and sex-matched healthy controls from the same geographic area were enrolled. SSc patients fulfilled the 2013 classification criteria for SSc of the American College of Rheumatology and the European League Against Rheumatism (ACR/EULAR) [27]. Patients under the age of 18 years, those with current or previous malignancy, and pregnant or breastfeeding women were excluded from the study. Controls were screened for additional exclusion criteria: history of immune-mediated disease (including organ-specific autoimmune disorders, e.g., autoimmune thyroid disease). The two main subtypes of SSc—lcSSc and dcSSc—were considered in equal proportions, with 26 patients each. Clinical data regarding disease manifestations and comorbidities were retrieved from medical records at the time of sampling.

All participants were included according to the Helsinki Declaration and its later amendments. The study was approved by the Hungarian Scientific Research Council Ethical Committee (No. V/418-1/2021/EKU). Informed consent was obtained from all individual participants included in the study.

### 2.2. Peripheral Blood Mononuclear Cell Separation and RNA Isolation

Venous peripheral blood samples were collected (10 mL) in vacuum collection tubes containing EDTA (BD Vacutainer K2EDTA; Becton-Dickinson, Franklin Lakes, NJ, USA). PBMCs were separated by Ficoll gradient centrifugation. Total RNA was extracted from PBMC using Trizol reagent (Invitrogen, Carlsbad, CA, USA), according to the manufacturer’s protocol, on the day of blood sampling. RNA quality was checked on an Agilent Bioanalyzer 2100 (Agilent Technologies, Palo Alto, CA, USA); all samples had a 28S/18S ratio between 1.5 and 2.0, and the RNA integrity number was >7.

### 2.3. Small RNA-Seq Library Preparation

Small RNA-Seq libraries were generated from 1 μg total RNA using the NEBNext Multiplex Small RNA Perp Set for Illumina (1–48) 96 rxn kit (New England BioLabs, Ipswich, MA, USA), according to the manufacturer’s instructions. The fragment size distribution and molarity of libraries were checked on an Agilent BioAnalyzer DNA1000 chip (Agilent Technologies, Santa Clara, CA, USA). Then, a single-read 50 bp sequencing run was performed on an Illumina NextSeq 500 instrument (Illumina, San Diego, CA, USA) and an average of 8 million of raw reads per sample generated. The entire data set has been deposited in the Sequence Read Archive database of the NCBI under BioProject ID: PRJNA1477708 (https://www.ncbi.nlm.nih.gov/bioproject/PRJNA1477708, accessed on 2 July 2026).

### 2.4. Small RNA-Seq Data Analysis

Raw sequencing data (fastq) was mapped to the human reference genome version GRCh38 assembly GCA_000001405.15, and miRBase v22 annotation was used for annotating miRNAs. The Novoalign v3.00.05 aligner was used for the mapping; 90% of the raw reads were mapped, and BAM files were generated. Downstream analysis was performed using StrandNGS software v3.4 (Strand Life Sciences Pvt. Ltd., Bangalore, India; www.strand-ngs.com, accessed on 26 May 2026). BAM files were imported into the software, and the integrated DESeq algorithm was used for quantification and normalization. The Mann–Whitney U-test was used to identify differentially expressed miRNAs (DEMs) between conditions.

### 2.5. mRNA Library Preparation and Sequencing

A total of 250 ng of total RNA was used for library preparation. RNA-Seq libraries were prepared by using the MGIEasy RNA Library Prep Set V3.0 (MGI Tech, Shenzhen, China), according to the manufacturer’s protocol. Briefly, polyA RNAs were purified by oligo-dT conjugated magnetic beads and fragmented at 94 °C for 8 min; then, first-strand cDNA synthesis was immediately performed, which was followed by second-strand synthesis, and double-stranded cDNA was generated. After the magnetic-bead-based purification step, end-repair and A-tailing steps were performed; then, double-stranded oligonucleotide adapters were ligated. Ligated products were purified and amplified by using an adapter-specific primer pair. After a purification step, double-stranded libraries were generated, quantified by a qubit fluorimeter, and the fragment size distribution was checked on a BioAnalyzer DNA 1000 chip (Agilent Technologies, Santa Clara, CA, USA). Double-stranded libraries derived from different samples were pooled together in an equal amount. Pooled libraries were denatured, single-strand circularized, and digested, and after a purification step, a single-stranded DNA (ssDNA) library was generated. This library was used to make DNA NanoBall (DNB) by rolling-circle amplification. The DNBs then underwent high-throughput sequencing on an MGI DNBSEQ G400 instrument (MGI Tech, Shenzhen, China). Single-end 100-cycle sequencing runs were performed using the DNBSEQ-G400RS high-throughput Sequencing Set (FCL SE100; Standard-MPS) reagent, and an average of 22 million of raw reads were generated. The sequencing data have been deposited in the Sequence Read Archive database of the NCBI under BioProject ID: PRJNA1477707 (https://www.ncbi.nlm.nih.gov/bioproject/PRJNA1477707, accessed on 2 July 2026). Benjamini–Hochberg FDR-adjusted q-values were calculated for all expressed miRNAs tested in each differential-expression comparison. Complete miRNA result tables, including nominal *p*-values and corresponding FDR-adjusted q-values, are provided in Supplementary Tables S1–S5.

### 2.6. mRNA-Seq Data Analysis

Fastq files were aligned to the human reference genome version GRCh38 assembly GCA_000001405.15 using the HISAT2 v2.1 alignment program, and gene annotation was performed using the Ensembl Genes v87 annotation database. A total of 95% of the raw reads were mapped, and BAM files were generated. Downstream analysis was performed using StrandNGS software v3.4 (Strand Life Sciences Pvt. Ltd., Bangalore, India; www.strand-ngs.com). BAM files were imported, and the integrated DESeq algorithm of the software was used for quantification and normalization. The Mann–Whitney unpaired test with the Benjamini–Hochberg false discovery rate (FDR) for multiple testing correction was used to determine differentially expressed genes (DEGs) between conditions, with an FDR < 0.05 considered statistically significant. Gene Ontology (GO), Kyoto Encyclopedia of Genes and Genomes (KEGG), and Reactome functional enrichment analyses and visualizations were performed using R version 4.5.1.

## 3. Results

### 3.1. Demographic and Clinical Characteristics of SSc Patients

A total of fifty-two SSc patients were included in the study. Ratios of women and men were representative for the disease: 42 women (81%) and 10 men (19%). Thirty-five patients were diagnosed with SSc-ILD, while 17 patients presented without lung manifestations. Characteristics of the patient cohort are summarized in Table 1.

### 3.2. SSc Patients vs. Healthy Controls

Altogether, 58 miRNAs showed significantly differential expression in SSc patients compared to controls. Among these miRNAs, 33 were significantly upregulated and 25 downregulated.

**Figure 1:**
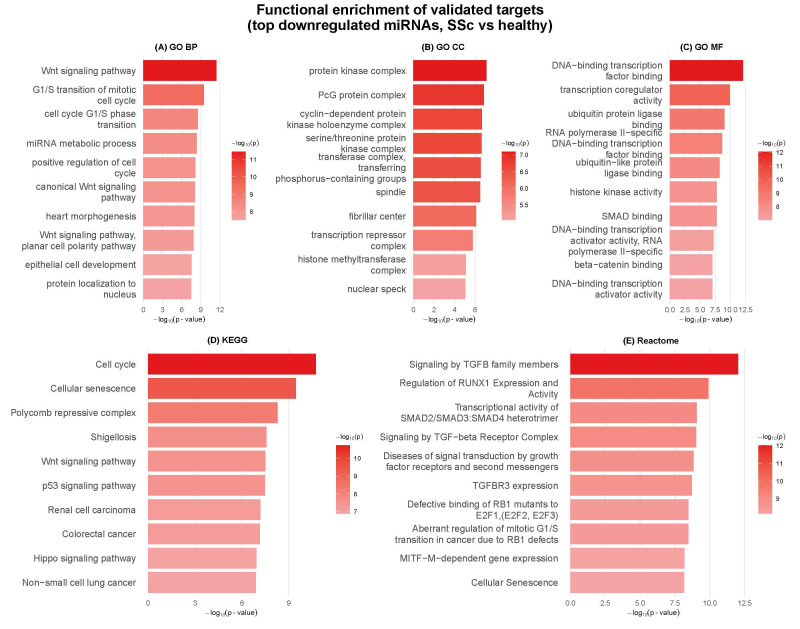
(**A**–**E**) Functional enrichment analysis of validated targets of top 8 downregulated miRNAs (FC < −1.5, *p* < 0.05) in SSc compared to controls. Gene Ontology (GO), KEGG and Reactome pathway enrichment analyses were performed on experimentally validated target genes of the top downregulated miRNAs identified in SSc compared to healthy controls. Target genes were derived from miRTarBase and TarBase, and only high-confidence interactions supported by both databases were retained. (**A**–**C**) GO enrichment analysis showing the top 10 significantly enriched terms (*p* < 0.05) for Biological Process (BP), Cellular Component (CC), and Molecular Function (MF), respectively. (**D**) KEGG pathway enrichment analysis of validated target genes, displaying the top 10 enriched pathways. (**E**) Reactome pathway enrichment analysis of validated target genes, showing the top enriched pathways. Bars length represents significance of enrichment as −log_10_(*p*-value), with darker color intensity indicating stronger statistical significance. Terms are ordered by increasing significance.

Nine downregulated miRNAs reached the significance of fold change (FC) < −1.5 and *p* < 0.05 and were selected for downstream functional annotation of their experimentally validated target genes (Table 2). Validated targets of these top downregulated miRNAs were retrieved from miRTarBase and TarBase, and only targets supported by both databases were retained to increase confidence in the target set. This resulted in 897 unique high-confidence validated target genes, which were used for GO and functional enrichment analyses (Figure 1A–E). GO Biological Process (BP) analysis of this validated target set showed significant enrichment of pathways related primarily to Wnt signaling. GO Cellular Component (CC) analysis highlighted enrichment in protein kinase complexes, cyclin-dependent kinase holoenzyme complexes, and nuclear-associated structures. GO Molecular Function (MF) analysis indicated enrichment of DNA-binding transcription factor activity, transcription coregulator activity, ubiquitin ligase binding, and SMAD binding. KEGG pathway analysis demonstrated significant enrichment in Wnt signaling. Reactome pathway analysis revealed enrichment in TGFβ signaling, SMAD2/3-SMAD4 transcriptional activity, RUNX1 regulation, and related signal transduction pathways.

**Figure A1:**
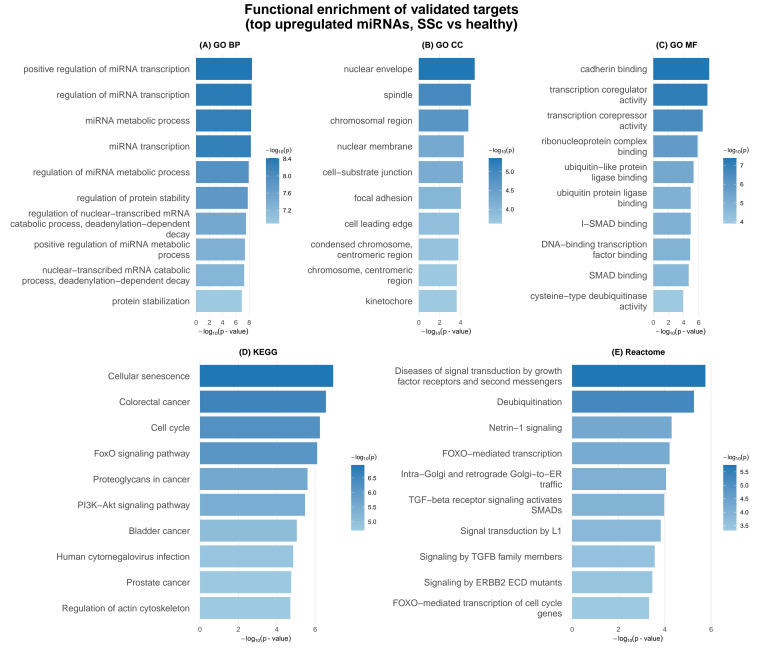
(**A**–**E**) Functional enrichment analysis of validated targets of top 11 upregulated miRNAs in SSc compared to controls (FC > 1.5, *p* < 0.05). Gene Ontology (GO), KEGG and Reactome pathway enrichment analyses were performed on experimentally validated target genes of the top upregulated miRNAs identified in SSc compared to healthy controls. Target genes were derived from miRTarBase and TarBase, and only high-confidence interactions supported by both databases were retained. (**A**–**C**) GO enrichment analysis showing the top 10 significantly enriched terms (*p* < 0.05) for Biological Process (BP), Cellular Component (CC), and Molecular Function (MF), respectively. (**D**) KEGG pathway enrichment analysis of validated target genes, displaying the top 10 enriched pathways. (**E**) Reactome pathway enrichment analysis of validated target genes, showing the top enriched pathways. Bars length represents significance of enrichment as −log_10_(*p*-value), with darker color intensity indicating stronger statistical significance. Terms are ordered by increasing significance.

Experimentally validated targets of the top upregulated miRNAs (11 miRNAs) reaching the significance of FC > 1.5 and *p* < 0.05 have been collected from the miRTarBase and TarBase databases. Only targets supported by both databases were retained; this resulted in 683 unique high-confidence validated target genes, which were used for GO, KEGG, and Reactome enrichment analyses. GO enrichment analysis of this validated target set revealed significant overrepresentation of terms associated with miRNA regulation and gene expression control. KEGG and Reactome analyses further supported the involvement of these genes in regulatory and signaling pathways. The list of top upregulated miRNAs is shown in Table 2, and the top 10 GO, KEGG, and Reactome enrichment results for their validated targets are presented in Figure A1A–E.

### 3.3. Subtype-Specific Investigation of Differentially Expressed miRNAs

**Figure 2:**
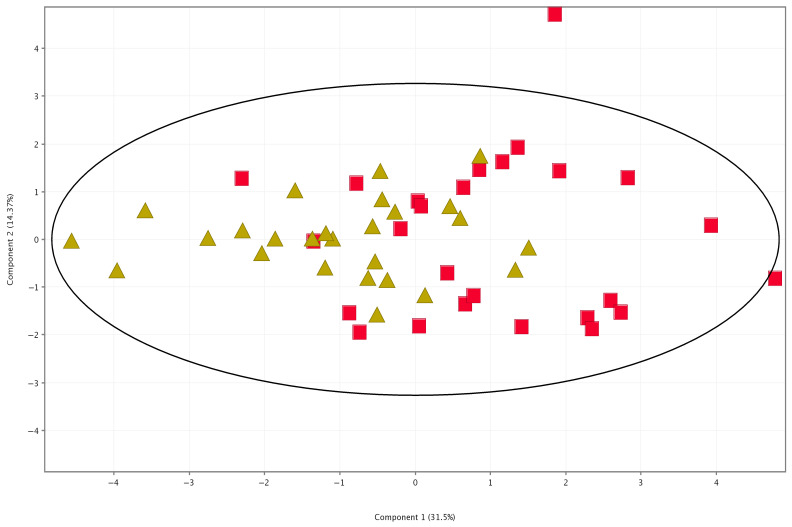
Principal Component Analysis (PCA) plot of diffuse cutaneous (dcSSc, *n* = 26) and limited cutaneous (lcSSc, *n* = 26) patients based on their miRNA expression profiles. The first principal component accounts for 31.5% and the second principal component accounts for 14.37% of the total variance. Yellow triangles represent lcSSc patients, while red squares indicate dcSSc patients. Each point corresponds to an individual patient. The ellipse denotes the overall data distribution.

To evaluate the differential diagnostic potential of miRNAs regarding the two main subtypes of the disease, we collected samples from lcSSc and dcSSc patients in equal ratios (26 patients from each). By analyzing the altered miRNA background between the two subgroups, we identified 10 differentially expressed miRNAs: four were significantly upregulated and six were significantly downregulated in dcSSc compared to the lcSSc group (FC > 1 or FC < −1, *p* < 0.05). Principal component analysis (PCA) showed a partial separation between dcSSc and lcSSc samples, with the first and second principal components accounting for 31.5% and 14.37% of the total variance, respectively. This may suggest subtype-related differences in PBMC miRNA expression profiles between dcSSc and lcSSc patients (Figure 2).

**Figure 3:**
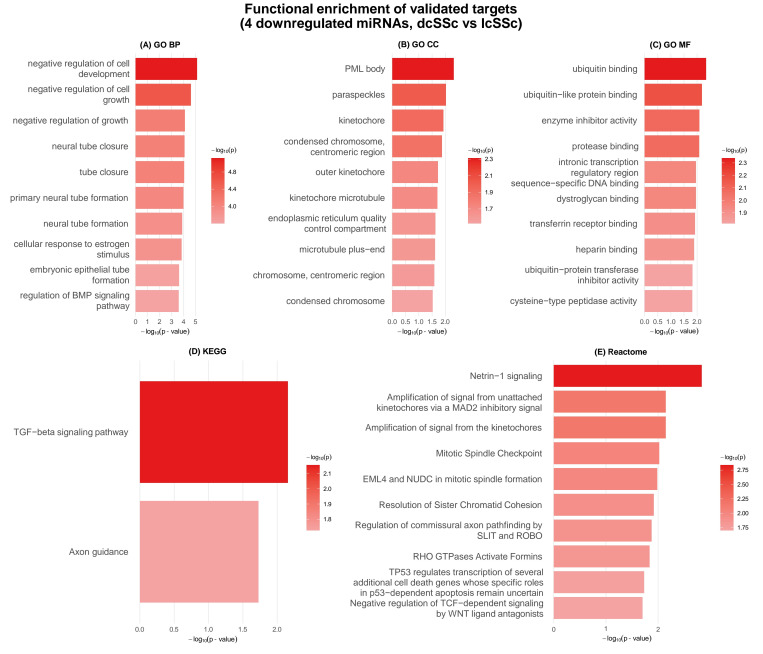
(**A**–**E**) Functional enrichment analysis of validated targets of top 4 downregulated miRNAs comparing diffuse cutaneous (dcSSc, *n* = 26) and limited cutaneous (lcSSc, *n* = 26) patients (FC < −1.5, *p* < 0.05). Gene Ontology (GO), KEGG and Reactome pathway enrichment analyses were performed on experimentally validated target genes of the top downregulated miRNAs identified in dcSSc compared to lcSSc patients. Target genes were derived from miRTarBase and TarBase, and only high-confidence interactions supported by both databases were retained. (**A**–**C**) GO enrichment analysis showing the top 10 significantly enriched terms (*p* < 0.05) for Biological Process (BP), Cellular Component (CC), and Molecular Function (MF), respectively. (**D**) KEGG pathway enrichment analysis of validated target genes, displaying the top 10 enriched pathways. (**E**) Reactome pathway enrichment analysis of validated target genes, showing the top enriched pathways. Bars length represents significance of enrichment as −log_10_(*p*-value), with darker color intensity indicating stronger statistical significance. Terms are ordered by increasing significance.

To identify subtype-specific miRNA target genes, the four top downregulated miRNAs (hsa-miR-127-3p, hsa-miR-654-5p, hsa-miR-708-3p, hsa-miR-1468-5p) in the dcSSc versus lcSSc comparison were selected for further analyses based on FC criteria and statistical significance (FC < −1.5 and *p* < 0.05) (Table 3). Validated targets of these selected miRNAs were retrieved from miRTarBase and TarBase, and only targets supported by both databases were retained. This resulted in 19 unique high-confidence validated target genes. In addition, hsa-miR-4446-3p showed substantial downregulation in dcSSc compared with lcSSc (FC = −1.41, *p* = 0.0211), consistent with its significant downregulation in SSc patients compared with healthy controls (FC = −1.44, *p* = 0.0037) (Table 2). One significantly upregulated miRNA (*p* < 0.05), specifically, hsa-miR-2110, met the relaxed FC criteria of FC > 1.4 (FC = 1.47) (Table 3). GO, KEGG, and Reactome enrichment analyses of the 19 validated targets of the top downregulated miRNAs notably indicated TGFβ signaling and cell cycle regulation pathways relevant to fibrosis and immune dysregulation (Figure 3A–E).

### 3.4. SSc Patients with ILD and SSc Patients Without ILD

**Figure 4:**
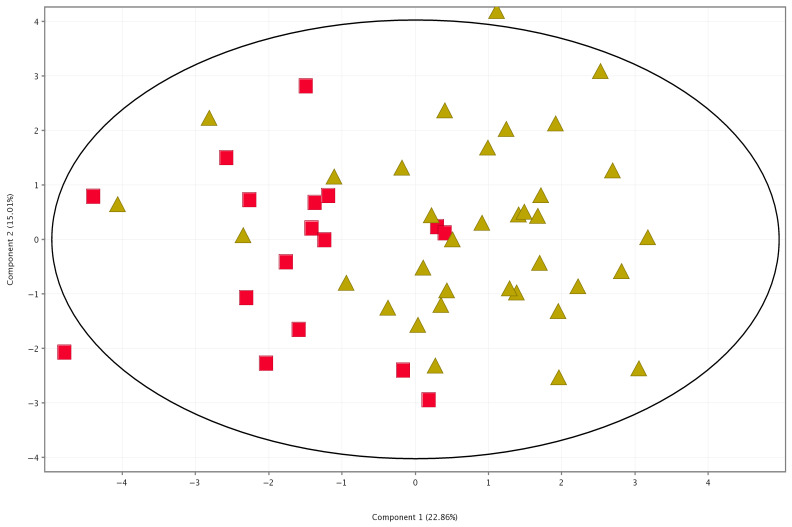
Principal Component Analysis (PCA) plot of SSc-ILD (*n* = 35) and SSc-noILD (*n* = 17) patients based on their miRNA expression profiles. The first and second principal components account for 22.86% and 15.01% of the total variance, respectively. Red squares represent SSc-noILD patients, while yellow triangles indicate SSc-ILD patients. Each point corresponds to an individual patient. The ellipse denotes the overall data distribution.

We aimed to study the molecular background of ILD, the main internal organ manifestation of the disease. To this end, we compared miRNA and mRNA expression profiles between 35 patients with SSc-ILD and 17 SSc patients without fibrotic lung involvement. In the presence of lung fibrosis, 12 miRNAs were significantly upregulated and 46 miRNAs significantly downregulated compared to non-ILD patients (FC > 1 or FC < −1, *p* < 0.05). PCA analysis of SSc-ILD and SSc-noILD samples showed a partial separation between the two groups based on miRNA expression profiles. The first and second principal components accounted for 22.86% and 15.01% of the total variance, respectively. Although SSc-ILD samples showed a tendency toward clustering along the first principal component, the overlap between groups indicates that this pattern should be interpreted as exploratory (Figure 4).

**Figure 5:**
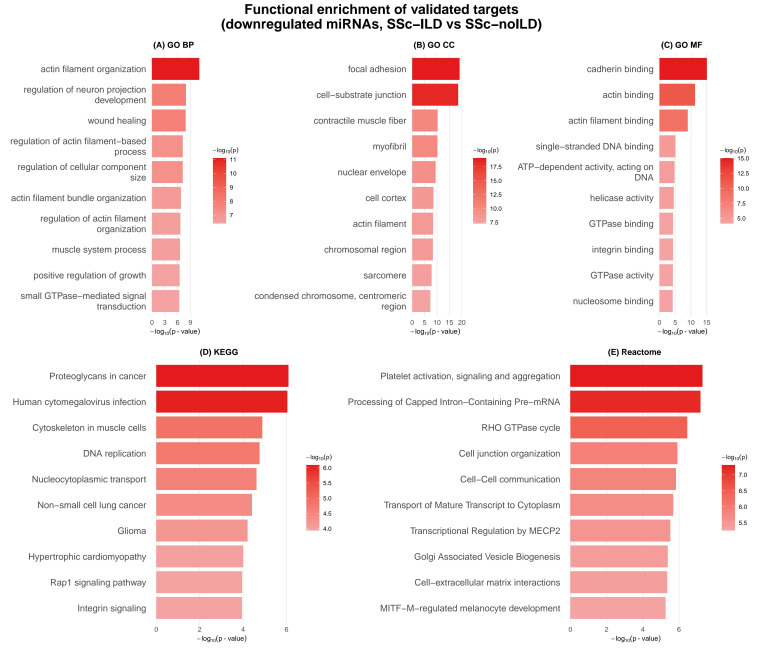
(**A**–**E**) Functional enrichment analysis of validated targets of top 11 downregulated miRNAs comparing SSc-ILD and SSc-noILD patients (FC < −1.5, *p* < 0.05). Gene Ontology (GO), KEGG and Reactome pathway enrichment analyses were performed on experimentally validated target genes of the top downregulated miRNAs identified in SSc-ILD compared to SSc-noILD patients. Target genes were derived from miRTarBase and TarBase, and only high-confidence interactions supported by both databases were retained. (**A**–**C**) GO enrichment analysis showing the top 10 significantly enriched terms (*p* < 0.05) for Biological Process (BP), Cellular Component (CC), and Molecular Function (MF), respectively. (**D**) KEGG pathway enrichment analysis of validated target genes, displaying the top 10 enriched pathways. (**E**) Reactome pathway enrichment analysis of validated target genes, showing the top enriched pathways. Bars length represents significance of enrichment as −log_10_(*p*-value), with darker color intensity indicating stronger statistical significance. Terms are ordered by increasing significance.

**Figure A2:**
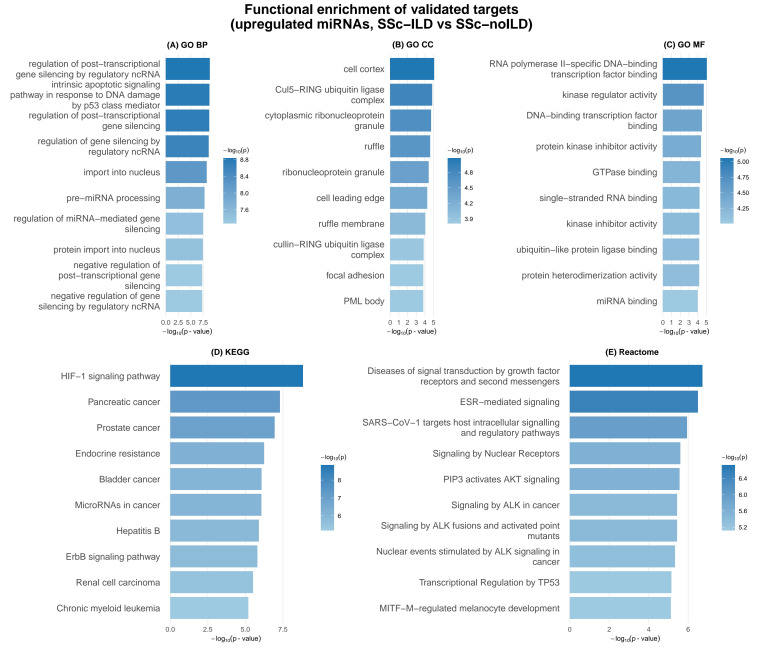
(**A**–**E**) Functional enrichment analysis of validated targets of top 9 upregulated miRNAs comparing SSc-ILD and SSc-noILD patients (FC > 1.3, *p* < 0.05). Gene Ontology (GO), KEGG and Reactome pathway enrichment analyses were performed on experimentally validated target genes of the top downregulated miRNAs identified in SSc-ILD compared to SSc-noILD patients. Target genes were derived from miRTarBase and TarBase, and only high-confidence interactions supported by both databases were retained. (**A**–**C**) GO enrichment analysis showing the top 10 significantly enriched terms (*p* < 0.05) for Biological Process (BP), Cellular Component (CC), and Molecular Function (MF), respectively. (**D**) KEGG pathway enrichment analysis of validated target genes, displaying the top 10 enriched pathways. (**E**) Reactome pathway enrichment analysis of validated target genes, showing the top enriched pathways. Bars length represents significance of enrichment as −log_10_(*p*-value), with darker color intensity indicating stronger statistical significance. Terms are ordered by increasing significance.

Twenty differentially expressed miRNAs were selected for further analysis in the SSc-ILD versus SSc-noILD comparison: 11 downregulated miRNAs (FC < −1.5, *p* < 0.05) and nine upregulated miRNAs (FC > 1.3, *p* < 0.05) (Table 4). Validated targets of these selected miRNAs were retrieved from miRTarBase and TarBase, and only targets supported by both databases were retained. This resulted in 95 unique high-confidence validated targets for the downregulated miRNAs and 213 unique high-confidence validated targets for the upregulated miRNAs. GO, KEGG, and Reactome enrichment analyses of the downregulated miRNA targets in SSc-ILD showed enrichment of terms related to cytoskeletal organization, cell adhesion, and RHO GTPase-related pathways, including focal adhesion and extracellular matrix (ECM) interactions (Figure 5A–E). Functional enrichment analysis of the upregulated miRNA targets indicated enrichment of pathways related to RNA-mediated gene silencing, transcriptional regulation, and HIF-1/PI3K-AKT signaling (Figure A2A–E). According to our gene expression analyses, *MMP9*, *MMP25*, *TNFRSF10C*, and *CXCR1* were significantly upregulated in PBMCs from SSc-ILD patients compared with the SSc-noILD group (FC > 2). In contrast, 28 immunoglobulin-related genes and one T-cell-associated gene (*TRDV2*) were downregulated in the SSc-ILD group (FC < −2). Altogether, these results suggest a shift toward innate immune activation and ECM remodeling, accompanied by relative depletion or redistribution of peripheral adaptive immune components.

### 3.5. SSc Patients with PAH Compared to SSc Patients Without PAH

**Figure 6:**
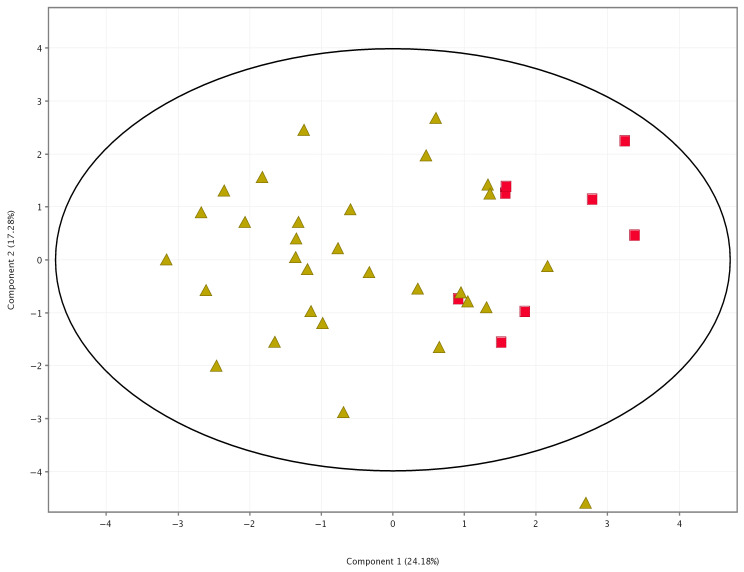
Principal Component Analysis (PCA) plot of SSc-PAH and SSc-noPAH patients based on their miRNA expression profiles. The first and second principal components account for 24.18% and 17.28% of the total variance, respectively. Yellow triangles represent SSc patients without PAH, while red squares indicate SSc-PAH patients. Each point corresponds to an individual patient. The ellipse denotes the overall data distribution.

Eight SSc-PAH patients were compared with SSc patients without PAH (SSc-noPAH). PCA analysis showed a modest separation between the groups along the first principal component, with the first and second principal components accounting for 24.18% and 17.28% of the total variance, respectively (Figure 6). Six differentially expressed miRNAs were further analyzed, four downregulated miRNAs (FC < −1.5, *p* < 0.05) and two upregulated miRNAs (FC > 1.5, *p* < 0.05) (Table 5).

**Figure 7:**
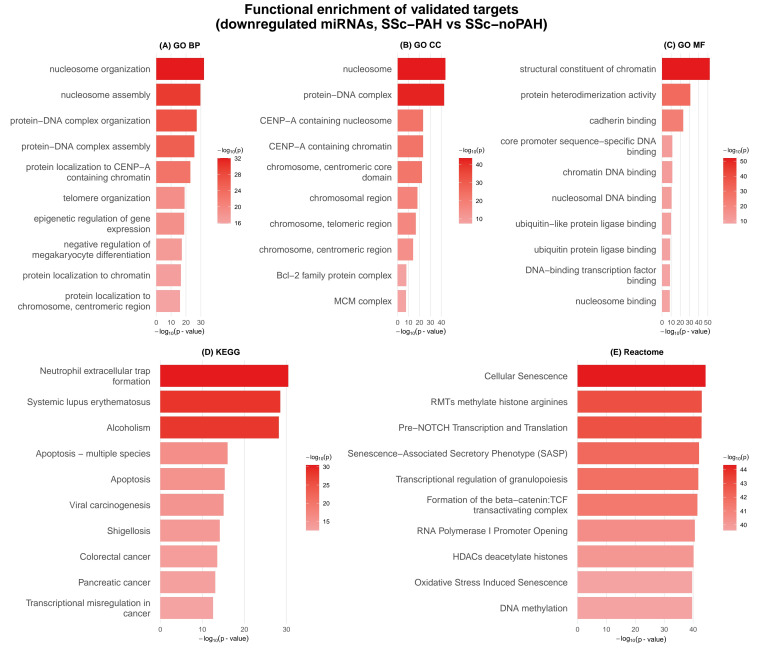
(**A**–**E**) Functional enrichment analysis of validated targets of top 4 downregulated miRNAs comparing SSc-PAH and SSc-noPAH patients (FC < −1.5, *p* < 0.05). Gene Ontology (GO), KEGG and Reactome pathway enrichment analyses were performed on experimentally validated target genes of the top downregulated miRNAs identified in SSc-PAH compared to SSc-noPAH patients. Target genes were derived from miRTarBase and TarBase, and only high-confidence interactions supported by both databases were retained. (**A**–**C**) GO enrichment analysis showing the top 10 significantly enriched terms (*p* < 0.05) for Biological Process (BP), Cellular Component (CC), and Molecular Function (MF), respectively. (**D**) KEGG pathway enrichment analysis of validated target genes, displaying the top 10 enriched pathways. (**E**) Reactome pathway enrichment analysis of validated target genes, showing the top enriched pathways. Bars length represents significance of enrichment as −log_10_(*p*-value), with darker color intensity indicating stronger statistical significance. Terms are ordered by increasing significance.

GO, KEGG, and Reactome enrichment analyses of the 75 unique high-confidence validated targets of downregulated miRNAs in the SSc-PAH comparison indicated enrichment of terms related to chromatin organization, epigenetic regulation, and apoptosis-related pathways (Figure 7A–E).

**Figure 8:**
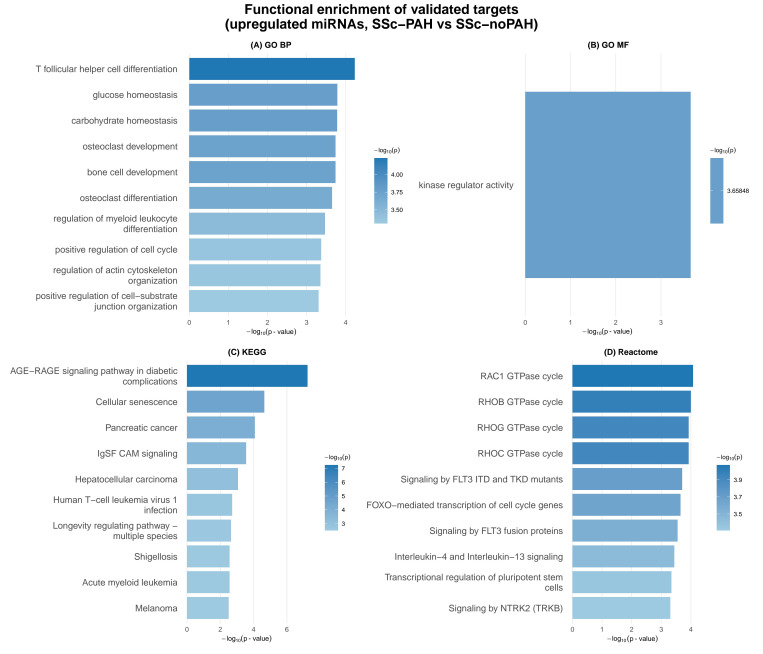

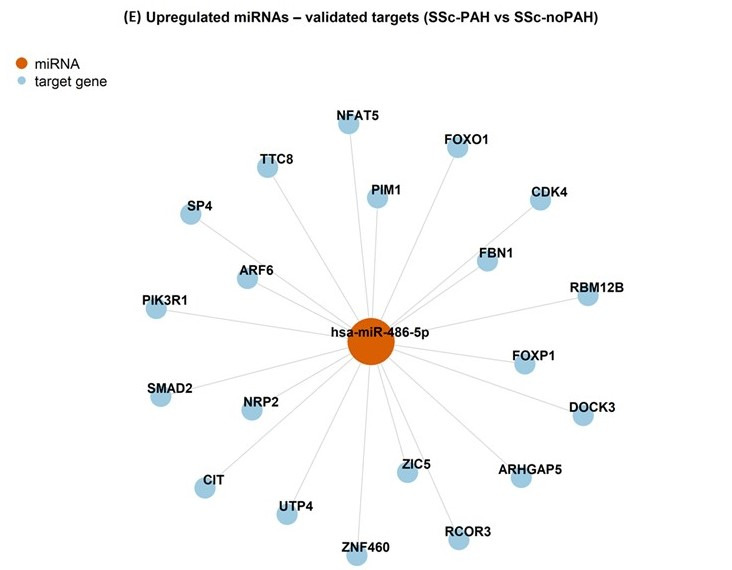
(**A**–**D**) Functional enrichment analyses of validated targets of top 2 upregulated miRNAs comparing SSc-PAH and SSc-noPAH patients (FC > 1.5, *p* < 0.05). Gene Ontology (GO), KEGG and Reactome pathway enrichment analyses were performed on experimentally validated target genes of the top upregulated miRNAs identified in SSc-PAH compared to SSc-noPAH patients. Target genes were derived from miRTarBase and TarBase, and only high-confidence interactions supported by both databases were retained. (**A**–**B**) GO enrichment analysis showing the top 10 significantly enriched terms (*p* < 0.05) for Biological Process (BP), Cellular Component (CC), and Molecular Function (MF), respectively. (**C**) KEGG pathway enrichment analysis of validated target genes, displaying the top 10 enriched pathways. (**D**) Reactome pathway enrichment analysis of validated target genes, showing the top enriched pathways. Bars length represents significance of enrichment as −log_10_(*p*-value), with darker color intensity indicating stronger statistical significance. Terms are ordered by increasing significance. (**E**) miRNA–target interaction network of upregulated miRNAs in SSc-associated pulmonary arterial hypertension (SSc-PAH). Network visualization of validated target genes of upregulated miRNAs in SSc-PAH compared to SSc without PAH. The central node represents hsa-miR-486-5p (orange), while connected nodes (blue) indicate experimentally validated target genes. Edges represent miRNA–target interactions.

Functional enrichment analysis of the 20 unique high-confidence validated targets of upregulated miRNAs in the SSc-PAH comparison indicated enrichment of terms related to immune differentiation, metabolic pathways, and RHO GTPase-related signaling (Figure 8A–D). The miRNA–target interaction network highlighted hsa-miR-486-5p as a central hub regulator in SSc-PAH, interacting with multiple validated targets involved in cell cycle regulation (*CDK4*), transcriptional control (*FOXO1*, *FOXP1*, *NFAT5*), ECM organization (*FBN1*), and cytoskeletal signaling (*ARHGAP5*, *DOCK3*) (Figure 8E).

### 3.6. Responsive (R) and Non-Responsive (NR) SSc-ILD Patients

**Figure 9:**
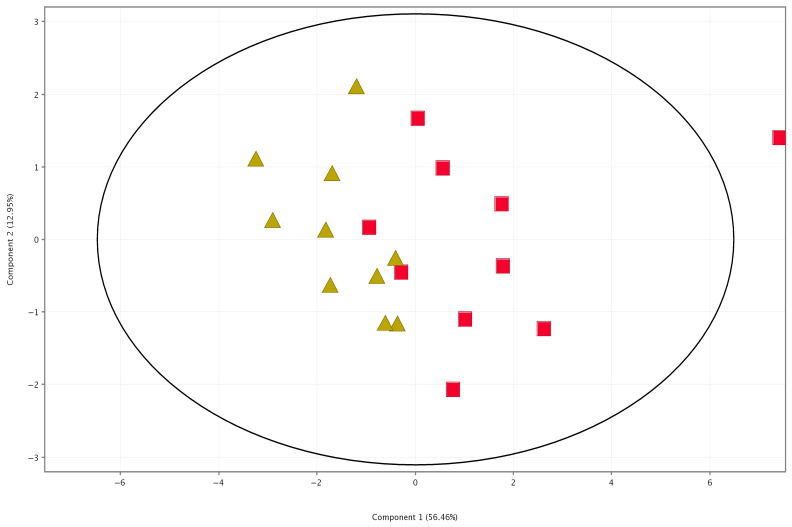
Principal component analysis (PCA) plot of responsive (R) and non-responsive (NR) SSc-ILD patients based on their miRNA expression profiles. The first and second principal components account for 56.46% and 12.95% of the total variance, respectively. Yellow triangles represent responsive patients, while red squares indicate non-responsive patients. Each point corresponds to an individual patient. The ellipse denotes the overall data distribution.

ILD is typically managed with immunosuppressant and/or antifibrotic therapies, and disease status is routinely assessed using a combination of forced vital capacity (FVC), high-resolution computed tomography (HRCT), and diffusion capacity testing (DLCO). We selected 20 SSc-ILD patients on stable immunosuppressive/antifibrotic therapy for at least one year and distributed them into two well-defined groups (10 Responsive and 10 Non-responsive) based on comprehensive clinical evaluation over the last 5 years. The non-responsive (NR) group was defined by clinically meaningful disease progression, exhibiting a relative FVC decline of ≥10%, or a 5–9% FVC decline coupled with radiographic worsening on HRCT, alongside DLCO deterioration or remaining under DLCO < 70% with less than 15% improvement. Conversely, patients were considered responsive (R) if they demonstrated stable or improving FVC, HRCT stabilization, and >15% improvement or halting of prior deterioration in DLCO. Importantly, none of the selected 20 patients had confirmed PAH; therefore, PAH was not present in either subgroup and could not have acted as a confounding factor influencing the transcriptomic data. PCA analysis of these 20 SSc-ILD patients showed a tendency toward separation between R and NR patients along the first principal component, which accounted for 56.46% of the total variance. The second principal component accounted for 12.95% of the variance. Given the limited sample size, this pattern should be interpreted as exploratory (Figure 9).

In the R group, two miRNAs were significantly upregulated and seven miRNAs were significantly downregulated compared to the NR group (FC > 1.5 or FC < −1.5 and *p* < 0.05) (Table 6). Notably, we found significant upregulation of hsa-miR-2110 in dcSSc patients compared to the lcSSc subtype (Table 3), while significant upregulation of hsa-miR-194-3p and hsa-miR-941 was observed in SSc-ILD vs. SSc-noILD patients (Table 4). Three significantly expressed miRNAs, belonging to the same miRNA family (let-7 family), showed a unidirectional differentiation (hsa-let-7a-5p, hsa-let-7f-5p, and hsa-miR-98-5p with FC values of −1.14, −1.17, and −1.20, respectively), suggesting a synergic impact on their predicted targets, including several types of collagen molecules (*COL24A1*, *COL4A2*, *COL14A1*, *COL9A1*), according to the TargetScanHuman 7.2 database. Our gene expression analysis revealed significant downregulation of *TRAJ27*, *TRBV7-6*, and *TRDV1* genes (FC < −2) in NR SSc-ILD patients, suggesting reduced peripheral T-cell receptor expression, potentially reflecting T-cell redistribution to the lung or functional exhaustion.

### 3.7. Differentially Expressed Genes (DEGs) in SSc Patients Compared to Healthy Controls

**Figure 10:**
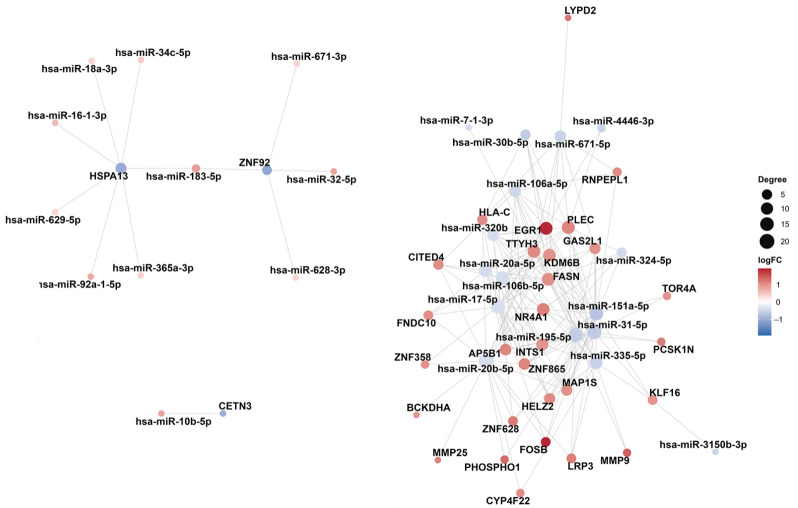
Differentially expressed miRNA–mRNA network. The network was generated using FDR-corrected differentially expressed mRNAs and differentially expressed miRNAs selected based on unadjusted *p*-value and fold-change criteria. A total of 180 unique inverse miRNA–mRNA pairs were identified. Node coloring reflects logFC values, with red indicating higher and blue indicating lower expression.

**Figure A3:**
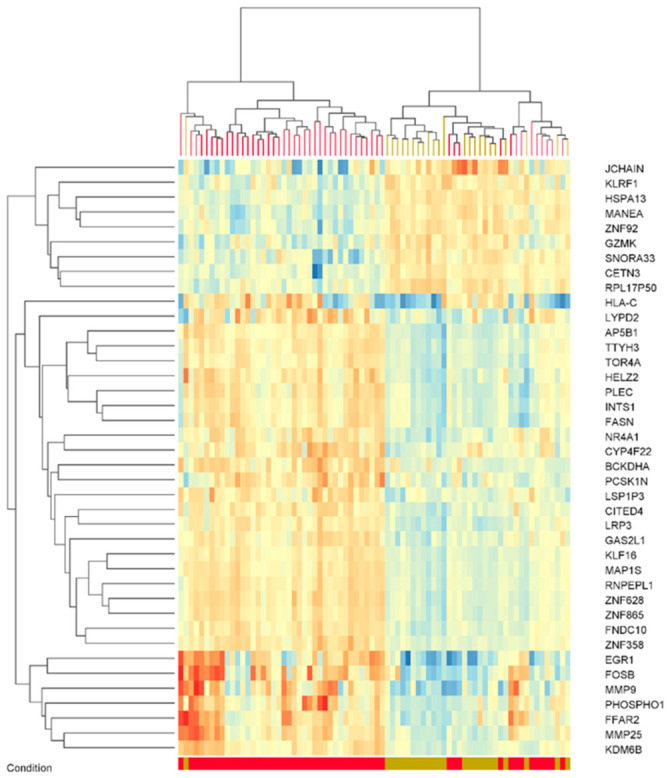
Differentially expressed mRNA profile comparing SSc patients and healthy controls (FC < –2 or FC > 2, FDR < 0.05). Heatmap representing the normalized expression levels of differentially expressed mRNAs between patients with systemic sclerosis (SSc) and healthy controls. Rows represent individual mRNAs, while columns correspond to individual samples. Sample groups are indicated by the annotation bar: SSc samples are denoted in red and controls in yellow. Expression levels are scaled and visualized using a color gradient, where red indicates higher expression and blue indicates lower expression relative to the mean.

In total, we identified 6610 DEGs by mRNA sequencing of the SSc and control samples (52 SSc patients and 24 healthy controls) using the Mann–Whitney U-test (*p* < 0.05). Thirty-one upregulated and nine downregulated DEGs remained significant after Benjamini–Hochberg FDR multiple testing correction (Figure A3). Using the miRTarBase and TarBase databases, we identified 180 unique, experimentally validated, inverse miRNA–mRNA pairs between the 58 DEMs identified in the SSc vs. control comparison, based on unadjusted *p*-value and fold-change thresholds and the 40 FDR-corrected DEGs (Figure 10).

**Figure 11:**
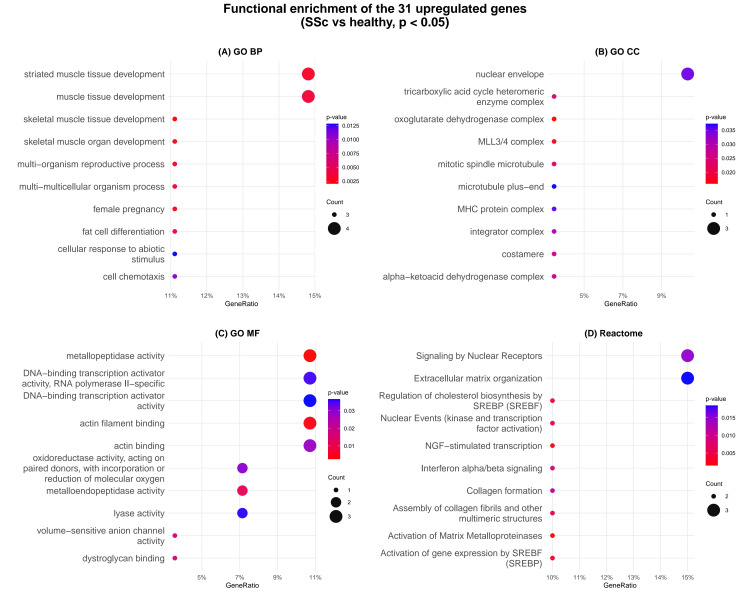
(**A**–**D**) Functional enrichment analyses of the 31 upregulated genes (SSc vs. healthy controls, FDR < 0.05). (**A**) Gene Ontology (GO) Biological Process (BP), (**B**) Cellular Component (CC), (**C**) Molecular Function (MF), and (**D**) Reactome pathway enrichment analyses are shown. For each panel, the top 10 significantly enriched terms are displayed, ranked primarily by the number of genes involved (Count) and secondarily by statistical significance (*p*-value). The x-axis represents the gene ratio (proportion of input genes associated with a given term), while dot size reflects the number of genes contributing to each term. Color intensity corresponds to the nominal *p*-value of enrichment. Only terms with *p* < 0.05 are included.

Functional enrichment analyses of the 31 FDR-significant upregulated DEGs in PBMCs showed enrichment of biological processes related to muscle tissue development, skeletal muscle differentiation, and cell chemotaxis. MF categories were dominated by metallopeptidase activity, suggesting potential involvement in extracellular matrix remodeling. Reactome pathway analysis indicated enrichment of ECM organization, collagen formation, and interferon signaling, consistent with pathways related to fibrosis and immune activation (Figure 11A–D).

**Figure 12:**
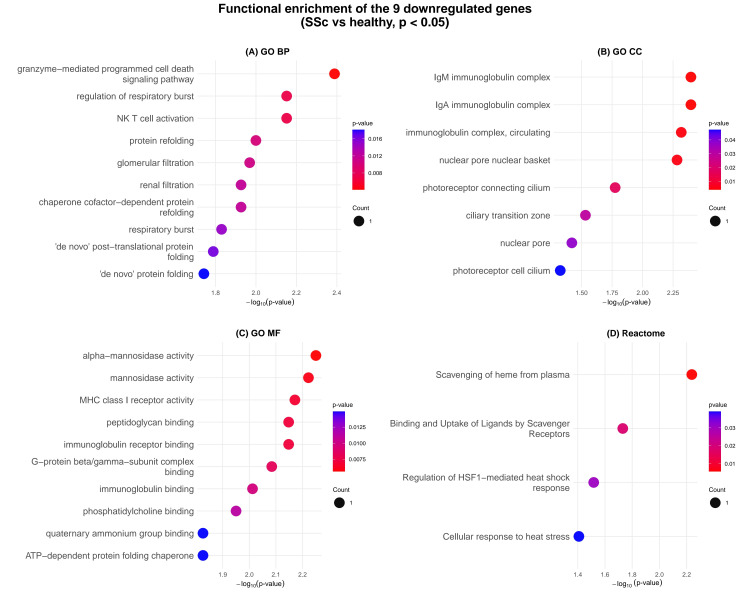
(**A**–**D**) Functional enrichment analyses of the 9 downregulated genes (SSc vs. healthy controls, FDR < 0.05). (**A**) Gene Ontology (GO), Biological Process (BP), (**B**) Cellular Component (CC), (**C**) Molecular Function (MF), and (**D**) Reactome pathway enrichment analyses are shown. For each panel, the top 10 significantly enriched terms are displayed, ranked by statistical significance (*p*-value). The x-axis represents significance of enrichment as −log_10_(*p*-value), with color intensity indicating stronger statistical significance corresponding to the nominal *p*-value of enrichment. Terms are ordered by increasing significance. Only terms with *p* < 0.05 are included.

Functional enrichment analyses of the nine FDR-significant downregulated DEGs showed enrichment of biological processes related to granzyme-mediated programmed cell death, regulation of respiratory burst, NK T-cell activation, and protein folding. Cellular component categories included immunoglobulin complexes, circulating immune complexes, and nuclear pore-associated structures, while molecular function categories included mannosidase activity, immunoglobulin receptor binding, MHC class I receptor activity, and protein folding-related functions (Figure 12A–D). These findings suggest possible links to immune regulation and protein processing; however, given the small number of downregulated DEGs, they should be interpreted cautiously.

Overlap analysis revealed that 22 genes were shared between the validated targets (*n* = 897) of the top downregulated miRNAs (FC < −1.5, *p* < 0.05) and the 31 upregulated DEGs (FC > 2, FDR < 0.05) in our PBMC samples. This corresponds to 71% (22/31) of the upregulated FDR-significant DEGs, indicating a notable convergence between validated miRNA targets and observed transcriptional changes. These findings suggest that a large fraction of the observed DEGs may be directly regulated by the identified miRNAs, supporting a functional miRNA–mRNA regulatory axis in SSc PBMCs.

**Figure 13:**
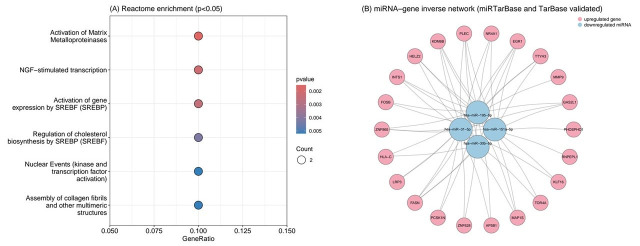
(**A**) Reactome enrichment analysis of the 22 upregulated genes. The 22 upregulated genes have been revealed with cross-section analysis of the 31 upregulated DEGs and the 897 miRTarBase- and TarBase-validated targets of the top 8 downregulated miRNAs in our study (SSc vs. healthy). (**B**) Inverse miRNA-gene regulatory network of the 22 common genes (SSc vs. healthy). Network representation of interactions between downregulated miRNAs (blue nods) and upregulated target genes (pink nodes) identified in SSc patients compared to healthy controls. Edges indicate experimentally validated miRNA–target interactions from miRTarBase and TarBase.

Further analysis of the 22 overlapping genes indicated significant enrichment of Reactome pathways related to activation of matrix metalloproteinases, NGF-stimulated transcription, and SREBP-mediated gene expression and cholesterol biosynthesis. Additional enriched pathways included nuclear signaling events involving kinase and transcription factor activation, as well as assembly of collagen fibrils and multimeric ECM structures (Figure 13A). Owing to the limited gene number, GO and KEGG analyses did not yield significant enrichment.

Network analysis highlighted a many-to-many regulatory structure in which multiple miRNAs converged on shared inversely expressed targets involved in transcriptional regulation (e.g., EGR1, NR4A1, KLF16), ECM remodeling (e.g., MMP9, PLEC), immune-related processes (e.g., HLA-C), and metabolic regulation (e.g., FASN). These findings suggest that selected miRNA–mRNA relationships may contribute to regulatory changes affecting ECM remodeling, transcriptional control, immune-related pathways, and metabolic processes in SSc (Figure 13B).

## 4. Discussion

The pathomechanism of SSc is still highly investigated in several scientific studies. Early and accurate diagnosis is essential, while ongoing efforts to risk stratify patients have a central role in predicting both organ involvement and disease progression. miRNAs are potential biomarkers in systemic immunological diseases, such as SSc. In the present study, we applied high-throughput next-generation sequencing to screen both miRNA and mRNA expression profiles in PBMCs from SSc patients and healthy controls, as well as in clinically relevant SSc subgroups. We identified several miRNAs that may be linked to key biological pathways involved in fibroblast progression and collagen accumulation. These findings suggest that PBMC miRNA profiles may provide insight into systemic molecular alterations in SSc; however, further validation is required to determine their diagnostic, prognostic, or therapeutic relevance.

### 4.1. Dysregulated miRNAs in SSc Patients

Among the miRNAs upregulated in SSc patients compared with healthy controls, several have previously been implicated in immune regulation, vascular dysfunction, fibrosis, or related autoimmune conditions, including hsa-miR-16-1, hsa-miR-16-2, hsa-miR-27a-5p, hsa-miR-92a, hsa-miR-143, and hsa-miR-183, although data on their expression in PBMCs from SSc patients remain limited.

miR-16 family members have been reported in immune cells, particularly B cells, and their mature form, miR-16, has been linked to angiogenesis-related pathways through regulation of VEGF-associated signaling in endothelial cells [28,29]. Altered miR-16 expression has also been described in PBMCs from patients with other systemic autoimmune diseases, including primary Sjögren’s syndrome and systemic lupus erythematosus (SLE) [19]. In SSc, previous studies have reported altered expression of miR-16-5p, the typically dominant and more abundantly expressed strand, in serum samples and dermal fibroblasts, suggesting that miR-16-related regulation may differ across biological compartments and miRNA strands [30].

hsa-miR-27a-5p has been reported to be elevated in PBMCs from rheumatoid arthritis patients and reduced following tofacitinib treatment [31], whereas miR-27a-3p was previously found to be downregulated in whole blood from SSc patients [32]. These differences may reflect strand-specific and cell-type-specific regulation of miR-27a expression [33].

Similarly, hsa-miR-92a has been associated with vascular dysfunction and fibrotic processes in previous studies, including reports of elevated serum levels in SSc and functional links to TGFβ-related collagen accumulation in fibroblasts [34,35,36,37].

Upregulated serum levels of miR-143 have also been reported in SSc and correlated with forced vital capacity [38].

In vitro evidence suggests that hsa-miR-183 promotes CD4^+^ T-cell proliferation through the EGR1/PTEN/Akt pathway, with a potential contribution of miR-96 [39]. In our study, however, elevated hsa-miR-183-5p levels were identified; we did not observe a corresponding significant change in miR-96 expression in our PBMC dataset.

Notably, hsa-miR-4446-3p was downregulated both in SSc patients compared with healthy controls and in dcSSc patients compared with lcSSc patients. To our knowledge, hsa-miR-4446-3p has not previously been reported in SSc. Therefore, this finding may indicate a novel PBMC-associated miRNA signal related to disease status and clinical subtype; however, it should be considered exploratory and requires validation in independent cohorts. Evaluation of hsa-miR-4446-3p tissue expression using the miRNATissueAtlas 2025 database [40] shows variable expression across multiple organs, with the highest level in lung tissue and relatively elevated expression in bronchial samples, suggesting a potential role in pulmonary-tissue-specific regulatory processes.

### 4.2. Possible Connections Between Subtypes and ILD

Functional enrichment analysis of validated targets of downregulated miRNAs in the dcSSc versus lcSSc comparison indicated pathways related to TGFβ-associated signaling, cytoskeletal regulation, and cell cycle processes, which are relevant to fibrosis-related biology. Similarly, enrichment analysis of validated targets of downregulated miRNAs in the SSc-ILD versus SSc-noILD comparison highlighted processes related to actin filament organization, wound healing, and cellular growth regulation.

We observed upregulation of hsa-miR-2110 in dcSSc compared to the lcSSc subtype, as well as downregulation in therapy-responsive SSc-ILD patients compared to the non-responsive group. These findings suggest a possible association of hsa-miR-2110 with disease subtype and treatment-response-related molecular patterns in SSc. To our knowledge, hsa-miR-2110 has not previously been described in SSc; however, this observation remains exploratory and requires validation in independent cohorts.

hsa-miR-127-3p, hsa-miR-654-5p, and hsa-miR-708-3p were downregulated both in dcSSc compared with lcSSc and in SSc-ILD compared with SSc-noILD. This concordant pattern may indicate a shared PBMC miRNA signature associated with clinically more severe fibrotic manifestations of SSc. However, direct regulatory effects on profibrotic pathways cannot be inferred from these data alone. Previous studies have already demonstrated that circulating and tissue miRNA profiles differ across SSc disease subsets and correlate with organ involvement [41]. Although these three miRNAs have not previously been reported in SSc, hsa-miR-127-3p has been implicated in inflammatory regulation, including modulation of FcγRI/CD64-related macrophage responses during lung inflammation [42].

Our mRNA expression analysis showed increased expression of MMP9, MMP25, TNFRSF10C, and CXCR1 in PBMCs from SSc-ILD patients compared with SSc-noILD patients, suggesting a peripheral immune expression pattern consistent with innate immune activation and ECM-related remodeling processes. MMP9 and MMP25 are involved in matrix remodeling and leukocyte-associated inflammatory processes, while CXCR1 supports neutrophil-related inflammatory signaling [43,44,45]. In parallel, upregulation of *TNFRSF10C* may reflect increased resistance to apoptosis [46], a mechanism implicated in the persistence of profibrotic cells and the progression of tissue fibrosis [47]. Conversely, downregulation of immunoglobulin-related genes and TRDV2 may reflect altered peripheral adaptive immune signatures in SSc-ILD [48]. Together, these PBMC expression findings suggest that SSc-ILD is associated with immune activation and remodeling-related molecular features; however, tissue-level mechanisms and cellular redistribution require further validation in paired tissue or single-cell studies.

### 4.3. Possible Connections Between ILD and Therapeutic Responsiveness

We found significantly upregulated levels of hsa-miR-194-3p and hsa-miR-941 in SSc-ILD compared with SSc-noILD, and their expression levels were also higher in treatment-responsive SSc-ILD patients than in non-responsive SSc-ILD patients. These findings suggest that hsa-miR-194-3p and hsa-miR-941 may be associated not only with pulmonary involvement but also with treatment-response-related molecular patterns in SSc-ILD. This is consistent with the complex biology of SSc-ILD, involving epithelial/endothelial injury, immune dysregulation, aberrant repair, and fibroblast activation, and supports the need for molecular biomarkers that may complement conventional measures such as FVC, DLCO, and high-resolution computed tomography (HRCT) extent [49,50]. To our knowledge, neither hsa-miR-194-3p nor hsa-miR-941 has previously been reported in SSc. Previous experimental studies have linked miR-194-3p to fibroblast-related processes, including regulation of proliferation, migration, and TGFβ-associated fibroblast-to-myofibroblast conversion, and miR-194 has also been reported among miRNAs associated with fibrotic lung tissue [51,52,53]. hsa-miR-941 has been linked to TLR3-associated injury signaling, inflammatory and epigenetic regulation, and tissue-repair-related programs [54]. These literature data provide a biological context for our findings but do not establish direct functional involvement in SSc-ILD. Overall, our results suggest that hsa-miR-194-3p and hsa-miR-941 may represent PBMC-associated miRNA signals related to ILD and treatment-response status in SSc.

### 4.4. Observed Transcriptomic Signatures of Therapy Responsiveness in SSc-ILD

Integration of mRNA and miRNA differential expression data further supports that the therapeutic response in SSc-ILD may be associated with PBMC molecular patterns involving immune regulation and fibrosis-related pathways. In non-responders, downregulation of T-cell receptor-related genes, including TRAJ27, TRBV7-6, and TRDV1, may indicate reduced peripheral T-cell receptor signatures, which could reflect altered circulating T-cell composition, functional exhaustion, or redistribution of T-cell subsets, all of which have been described in SSc-ILD [48,55], although tissue-level mechanisms cannot be inferred directly from PBMC data alone. In parallel, the concomitant response-associated miRNA alterations point toward post-transcriptional regulation of ECM remodeling and targets related to immune signaling pathways. These patterns are consistent with a shift away from adaptive immune activity toward a more tissue-dominant, therapy-resistant disease phenotype.

### 4.5. Molecular Pathway Assessment of SSc-PAH

Our enrichment analyses suggest a potential dual regulatory pattern in SSc-PAH. Downregulated miRNAs were associated with validated targets involved in epigenetic and chromatin-related pathways, which may contribute to altered transcriptional regulation. This is consistent with previous studies demonstrating a role for epigenetic regulation and transcription factor activation in pulmonary vascular remodeling [56,57,58]. Conversely, upregulated miRNAs were linked to pathways involved in immune differentiation, metabolic processes, and cytoskeletal dynamics, which are relevant to PAH pathogenesis, including inflammation-driven vascular remodeling, metabolic reprogramming, and altered smooth muscle cell dynamics [58,59,60]. Given the limited size of the SSc-PAH subgroup, these findings should be considered exploratory and require validation in larger cohorts.

### 4.6. Integrated Analysis of SSc Patients vs. Healthy Controls

Functional enrichment analysis of the 31 FDR-significant upregulated genes in the SSc versus healthy control comparison suggested enrichment of pathways related to fibrosis, cytoskeletal organization, and immune activation. Enriched terms included ECM organization, collagen formation, metallopeptidase activity, actin-related functions, and interferon signaling, which are consistent with biological processes implicated in SSc pathogenesis. These findings may indicate that upregulated genes in PBMCs are associated with extracellular matrix remodeling and inflammatory signaling, reinforcing the suggested biological processes associated with our downregulated miRNA pattern, related primarily to Wnt signaling.

Functional enrichment analysis of the nine FDR-significant downregulated genes suggested possible involvement of immune effector functions and protein homeostasis-related pathways. Enriched terms included granzyme-mediated programmed cell death, NKT cell activation, respiratory burst, immunoglobulin complexes, receptor binding functions, and protein-folding-related processes. These findings may indicate altered immune regulation and cellular stress-response pathways in PBMCs from SSc patients. However, because this interpretation is based on a small number of downregulated DEGs, it should be considered exploratory and interpreted with caution.

Among the 31 upregulated FDR-significant genes, 22 overlapped with experimentally validated targets of downregulated miRNAs (71%). This high proportion suggests that a substantial subset of robustly upregulated PBMC genes may be linked to downregulated miRNAs through candidate inverse miRNA–mRNA relationships, consistent with a possible reduction in post-transcriptional repression.

Network analysis showed that the four downregulated miRNAs converged on the 22 shared upregulated targets. Several of these genes have previously been implicated in SSc or related autoimmune and fibrotic processes, including EGR1, MMP9, HLA-C, INTS1, and KDM6B [61,62,63,64,65,66]. Among the downregulated miRNAs included in this inverse miRNA–mRNA network, hsa-miR-30b-5p, hsa-miR-31-5p, and hsa-miR-195-5p have previously been linked to SSc or fibrosis-related processes. Lower serum levels of hsa-miR-30b-5p have been reported in SSc and were inversely correlated with skin involvement, while miR-30b was shown to repress PDGFR-β, COL1A1, and ASMA expression in dermal fibroblasts [67]. In contrast, elevated hsa-miR-31-5p levels have previously been detected in SSc skin tissues and fibroblasts, which may indicate compartment- or tissue-specific differences compared with our PBMC findings [68]. In addition, hsa-miR-195-5p has been reported to inhibit MMP9 expression, providing a further biological context for its inverse relationship with MMP9 in our network [69]. Together, these findings suggest that candidate inverse miRNA–mRNA relationships may contribute to a PBMC molecular signature involving fibrosis-related, immune, and metabolic pathways in SSc, although functional validation is required to confirm direct regulatory effects. Our transcriptomic analyses identified a limited set of robustly dysregulated mRNA features. In the mRNA-seq dataset, 6610 genes were nominally significant using *p* < 0.05, but only 40 genes remained significant after Benjamini–Hochberg FDR correction, including 31 upregulated and nine downregulated genes. This discrepancy reflects the impact of multiple testing correction in a heterogeneous clinical cohort, where biological variability and moderate effect sizes reduce statistical power. Thus, only genes with sufficiently strong and consistent expression changes remained significant after adjustment, while potentially relevant but more modest or cell-type-restricted signals may not reach the FDR threshold in bulk PBMC analysis [70,71,72,73].

Compared with mRNA profiles, miRNA expression profiles include fewer detectable features and often exert modest fine-tuning effects across multiple targets. In heterogeneous clinical cohorts and bulk sequencing settings, such effects may be further attenuated by cell-type composition and interindividual variability [74,75]. These factors likely contributed to the relatively limited number of detected miRNA changes in the present study and should be considered when interpreting the miRNA–mRNA integration results.

### 4.7. Limitations and Future Directions

Several limitations of this study should be acknowledged. First, the cross-sectional design precludes definitive conclusions regarding causality. While the identified miRNA and mRNA signatures and their associated pathways suggest potential mechanisms involved in SSc pathogenesis, longitudinal studies are necessary to determine whether these alterations represent causal drivers or secondary consequences of disease progression.

Second, our study relies entirely on a single discovery cohort. Although the study included well-characterized SSc patients and matched controls, and the overall cohort size provided sufficient statistical power for the primary case-control and major subtype analyses [76,77], smaller clinical subgroup analyses, particularly those involving PAH and treatment response, were limited by small sample size and were therefore underpowered. Furthermore, while the parallel sequencing of miRNA and mRNA from the exact same patient samples—combined with the use of experimentally validated target databases—provides strong internal consistency, the lack of an independent external validation cohort remains a limitation. Therefore, these results should be considered exploratory and hypothesis-generating and require validation in larger, independent cohorts.

Third, the potential impact of pharmacological treatment (including specific immunosuppressants, antifibrotics, and corticosteroids), as well as variations in disease duration, disease activity, systemic inflammation and smoking status, on gene and miRNA expression cannot be excluded. Importantly, because our cohort reflects real-world clinical practice rather than a randomized trial, treatment regimens were not homogeneous. Patients received various immunosuppressive/immunomodulatory agents and antifibrotic therapies according to standard-of-care guidelines. Therefore, these heterogeneous clinical variables may influence PBMC transcriptional profiles and act as potential confounding factors.

Fourth, for miRNA-seq analysis, differential expression was determined using unadjusted *p*-values combined with fold-change thresholds due to the limited number of detectable features and reduced statistical power. This approach was used in the context of an exploratory miRNA-seq analysis, where strict multiple testing correction may be overly conservative and may mask biologically relevant signals. A statistical limitation of the present study is that, although 16 miRNAs remained significant after Benjamini–Hochberg FDR correction in the SSc versus healthy control comparison, only seven of these also fulfilled the predefined fold-change criteria used for downstream prioritization, while miRNAs identified in the clinical subgroup analyses did not remain significant after strict FDR correction. Therefore, subgroup-associated miRNA candidates should be interpreted as exploratory and hypothesis-generating signals selected using nominal *p*-values and fold-change thresholds. The resulting miRNA–mRNA networks and target enrichment analyses provide a biological context for these candidates but do not replace independent statistical or functional validation. To improve statistical transparency, complete miRNA differential-expression result tables, including nominal *p*-values and FDR-adjusted q-values, are provided in Supplementary Tables S1–S5.

Finally, the use of PBMCs represents an additional limitation. PBMCs are a heterogeneous mixture of immune cell types, and differences in cell composition, including altered B-cell or T-cell proportions, may influence the observed expression patterns. Thus, changes in immunoglobulin- and T-cell-receptor-related genes should be interpreted with caution, as they may reflect cell-type composition rather than cell-intrinsic transcriptional changes. Future cell-type-specific or single-cell studies are needed to clarify the cellular origin of these findings.

## 5. Conclusions

The development of clinically utilizable gene expression assays has been attempted across numerous pathologies, including RA, psoriasis, and diverse malignancies. Beyond conventional biopsy-based diagnostics, the analysis of peripheral-blood-derived mononuclear cells offers a minimally invasive alternative for monitoring immune-mediated and autoimmune disorders. Integrating miRNA expression profiling with the evaluation of clinical parameters enables us to identify biological processes affected by inflammatory responses during disease progression. Mapping the connections between these transcriptomic alterations and clinical pathogenesis not only enhances our understanding of the underlying pathomechanisms of the disease but also facilitates the identification of novel biomarkers and potential therapeutic targets.

Interestingly, although our analyses were performed in PBMCs, we identified multiple fibrosis-related signatures, suggesting that these cells may capture not only immunological but also fibrotic processes, thereby providing a valuable, minimally invasive source of information for disease monitoring, prediction of progression, and assessment of therapeutic response in SSc.

## Figures and Tables

**Table 1:** Characteristics of SSc patients.

Parameters	SSc (*n* = 52)
Age (mean ± SD) (years)	59.1 ± 10.56
Women, *n* (%)	42 (80.8)
Disease characteristics	
Disease duration (mean ± SD) (years)	15.4 ± 11.24
Disease subtype	
Limited cutaneous, *n* (%)	26 (50.0)
Diffuse cutaneous, *n* (%)	26 (50.0)
Organ involvement	
Skin involvement, *n* (%)	52 (100.0)
ILD, *n* (%)	35 (67.3)
Cardiac involvement, *n* (%)	9 (17.3)
PAH, *n* (%)	8 (15.4)
Kidney involvement, *n* (%)	2 (3.8)
Treatment at blood sampling	
Low-dose glucocorticoid treatment, *n* (%)	6 (11.5)
Immunosuppressive/immunomodulatory therapy, *n* (%)	20 (38.5)
Antifibrotic therapy, *n* (%)	6 (11.5)
Smoking status	
Never smoker, *n* (%)	43 (82.7)
Former smoker, *n* (%)	6 (11.5)
Current smoker, *n* (%)	3 (5.8)
Disease activity/inflammation marker	
CRP at blood sampling, median [IQR], mg/L	2.64 [1.32–5.10]

Data are presented as mean ± standard deviation (SD), median [interquartile range, IQR], or number and percentage, as appropriate. Treatment exposure, smoking status and CRP levels were recorded at the time of blood sampling. Immunosuppressive/immunomodulatory therapy included methotrexate, mycophenolate mofetil, cyclophosphamide, tocilizumab, and rituximab. SSc: systemic sclerosis; ILD: interstitial lung disease; PAH: pulmonary arterial hypertension; CRP: C-reactive protein.

**Table 2:** Top differentially expressed miRNAs comparing SSc patients vs. healthy controls.

Mature miRNAs	FC Value	*p*-Value
Significantly downregulated miRNAs		
hsa-miR-30b-5p	−1.57	0.0003
hsa-miR-31-5p	−1.63	0.0038
hsa-miR-151a-5p	−1.55	0.0081
hsa-miR-195-5p	−1.55	0.0078
hsa-miR-652-3p	−1.53	0.0020
hsa-miR-1291	−1.72	0.0001
hsa-miR-3920	−1.57	0.02814
hsa-miR-4446-3p	−1.44	0.0037
hsa-miR-4772-5p	−1.68	0.0118
hsa-miR-10395-5p	−1.94	0.0008
Significantly upregulated miRNAs		
hsa-miR-10b-5p	1.79	0.0187
hsa-miR-16-1-3p	1.58	0.0002
hsa-miR-16-2-3p	1.34	0.035
hsa-miR-27a-5p	1.68	<0.0001
hsa-miR-32a-5p	1.84	<0.0001
hsa-miR-92a-1-5p	1.72	0.0001
hsa-miR-143-5p	1.71	0.0190
hsa-miR-183-5p	1.86	0.0227
hsa-miR-1294	1.74	0.0002
hsa-miR-3158-3p	1.68	0.0084
hsa-miR-3605-3p	1.60	0.0126
hsa-miR-3690	2.60	<0.0001
hsa-miR-5690	1.79	0.0031

The table contains 10 downregulated miRNAs (9 with FC < −1.5, *p* < 0.05 and 1 with FC < −1.4, *p* < 0.05), as well as 12 upregulated miRNAs (11 with FC > 1.5, *p* < 0.05 and 1 with FC > 1.3, *p* < 0.05), comparing SSc patients and healthy controls.

**Table 3:** Differentially expressed miRNAs comparing diffuse cutaneous (dcSSc, *n* = 26) vs. limited cutaneous (lcSS, *n* = 26) patients.

Mature miRNAs	FC Value	*p*-Value
hsa-miR-127-3p	−1.54	0.0404
hsa-miR-654-5p	−1.80	0.0386
hsa-miR-708-3p	−1.58	0.0478
hsa-miR-1468-5p	−1.53	0.0294
hsa-miR-4446-3p	−1.41	0.0211
hsa-miR-2110	1.47	0.0268

The table contains 5 downregulated miRNAs (FC < −1.4, *p* < 0.05) and 1 upregulated miRNA (FC > 1.4, *p* < 0.05) comparing diffuse cutaneous (dcSSc, *n* = 26) and limited cutaneous (lcSSc, *n* = 26) patients.

**Table 4:** Differentially expressed miRNAs comparing SSc-ILD vs. SSc-noILD patients.

Mature miRNAs	FC Value	*p*-Value
Significantly downregulated miRNAs		
hsa-miR-1-3p	−1.77	0.0439
hsa-miR-127-5p	−1.81	0.0143
hsa-miR-379-5p	−1.57	0.0415
hsa-miR-432-5p	−2.11	0.0020
hsa-miR-487b-3p	−1.67	0.0110
hsa-miR-654-5p	−1.77	0.0378
hsa-miR-708-3p	−1.74	0.0014
hsa-miR-708-5p	−1.72	0.0410
hsa-miR-3934-5p	−1.84	0.0039
hsa-miR-6501-5p	−1.53	0.0439
hsa-miR-6882-5p	−1.59	0.0011
Significantly upregulated miRNAs		
hsa-miR-125a-5p	1.52	0.0415
hsa-miR-181b-3p	1.39	0.0378
hsa-miR-194-5p	1.34	0.0343
hsa-miR-199b-5p	1.37	0.0031
hsa-miR-450a-5p	1.56	0.0311
hsa-miR-499a-5p	1.42	0.0180
hsa-miR-581	1.56	0.0126
hsa-miR-941	1.45	0.0187
hsa-miR-1226-3p	1.42	0.0231

The table contains 11 downregulated miRNAs (FC < −1.5, *p* < 0.05) and 9 upregulated miRNAs (FC > 1.3, *p* < 0.05) comparing SSc-ILD and SSc-noILD patients that were selected for further analyses.

**Table 5:** Differentially expressed miRNAs comparing SSc-PAH vs. SSc-noPAH patients.

Mature miRNAs	FC Value	*p*-Value
Significantly downregulated miRNAs		
hsa-miR-34a-5p	−1.66	0.0413
hsa-miR-671-5p	−1.55	0.0033
hsa-miR-942-5p	−1.81	0.0449
hsa-miR-10399-5p	−1.52	0.0081
Significantly upregulated miRNAs		
hsa-miR-486-5p	1.81	0.0413
hsa-miR-4433b-3p	1.99	0.0164

The table contains 4 downregulated miRNAs (FC < −1.5, *p* < 0.05) and 2 upregulated miRNAs (FC > 1.5, *p* < 0.05) comparing SSc-PAH and SSc-noPAH patients that were selected for further analyses.

**Table 6:** Differentially expressed miRNAs comparing responsive (R) vs. non-responsive (NR) SSc-ILD patients.

Mature miRNAs	FC Value	*p*-Value
Significantly downregulated miRNAs		
hsa-miR-2110	−1.59	0.0277
hsa-miR-6843-3p	−2.01	0.0142
Significantly upregulated miRNAs		
hsa-miR-194-3p	1.61	0.0120
hsa-miR-424-3p	1.52	0.0392
hsa-miR-542-3p	1.51	0.0494
hsa-miR-941	1.55	0.0470
hsa-miR-1296-5p	1.82	0.0340

The table contains 2 downregulated miRNAs with FC < −1.5, *p* < 0.05 and 5 upregulated miRNAs with FC > 1.5, *p* < 0.05 comparing responsive (R) and non-responsive (NR) SSc-ILD patients.

## Data Availability

The sequencing datasets generated during this study have been deposited in the NCBI Sequence Read Archive. The small RNA-seq dataset is available under BioProject ID PRJNA1477708: https://www.ncbi.nlm.nih.gov/bioproject/PRJNA1477708 (accessed on 2 July 2026). The mRNA-seq dataset is available under BioProject ID PRJNA1477707: https://www.ncbi.nlm.nih.gov/bioproject/PRJNA1477707 (accessed on 2 July 2026). Further data supporting the findings of this study are available from the corresponding author upon reasonable request.

## Supplementary Materials

The following supporting information can be downloaded at: https://www.mdpi.com/article/10.3390/biom16070994/s1, Table S1: Differentially expressed miRNAs in peripheral blood mononuclear cells (PBMCs) of systemic sclerosis (SSc) patients compared to healthy controls; Table S2: Differentially expressed miRNAs in peripheral blood mononuclear cells (PBMCs) of diffuse cutaneous systemic sclerosis (dcSSc) and limited cutaneous systemic sclerosis (lcSSc) patients; Table S3: Differentially expressed miRNAs in peripheral blood mononuclear cells (PBMCs) of SSc-ILD and SSc-noILD patients; Table S4: Differentially expressed miRNAs in peripheral blood mononuclear cells (PBMCs) of SSc-PAH and SSc-noPAH patients; Table S5: Differentially expressed miRNAs in peripheral blood mononuclear cells (PBMCs) of responsive (R) vs. non-responsive (NR) SSc-ILD patients.

## Abbreviations

The following abbreviations are used in this manuscript:

ACR	American College of Rheumatology
dcSSc	diffuse cutaneous systemic sclerosis
DEG	differentially expressed gene
DEM	differentially expressed miRNA
DLCO	diffusion capacity testing
DNB	DNA NanoBall
ECM	extracellular matrix
EULAR	European League Against Rheumatism
FC	fold change
FVC	forced vital capacity
GO	gene ontology
ILD	interstitial lung disease
KEGG	Kyoto Encyclopedia of Genes and Genomes
lcSSc	limited cutaneous systemic sclerosis
miRNA	micro-RNA
NR	non-responsive
PAH	pulmonary arterial hypertension
PBMC	peripheral blood mononuclear cell
PCA	Principal Component Analysis
R	responsive
RA	rheumatoid arthritis
SLE	systemic lupus erythematosus
SSc	systemic sclerosis
ssDNA	single-stranded DNA
